# High atmospheric carbon dioxide-dependent alleviation of salt stress is linked to RESPIRATORY BURST OXIDASE 1 (*RBOH1*)-dependent H_2_O_2_ production in tomato (*Solanum lycopersicum*)

**DOI:** 10.1093/jxb/erv435

**Published:** 2015-09-28

**Authors:** Changyu Yi, Kaiqian Yao, Shuyu Cai, Huizi Li, Jie Zhou, Xiaojian Xia, Kai Shi, Jingquan Yu, Christine Helen Foyer, Yanhong Zhou

**Affiliations:** ^1^Department of Horticulture, Zijingang Campus, Zhejiang University, 866 Yuhangtang Road, Hangzhou, 310058, P.R. China; ^2^Zhejiang Provincial Key Laboratory of Horticultural Plant Integrative Biology, 866 Yuhangtang Road, Hangzhou, 310058, P.R. China; ^3^Centre for Plant Sciences, School of Biology, Faculty of Biological Sciences, University of Leeds, Leeds, LS2 9JT, UK

**Keywords:** CO_2_ enrichment, Na^+^/K^+^ homeostasis, NADPH oxidase, reactive oxygen species, salt overly sensitive (SOS) signalling pathway, transpiration.

## Abstract

Plants acclimate rapidly to stressful environmental conditions. Increasing atmospheric CO_2_ levels are predicted to influence tolerance to stresses such as soil salinity but the mechanisms are poorly understood. To resolve this issue, tomato (*Solanum lycopersicum*) plants were grown under ambient (380 μmol mol^–1^) or high (760 μmol mol^–1^) CO_2_ in the absence or presence of sodium chloride (100mM). The higher atmospheric CO_2_ level induced the expression of *RESPIRATORY BURST OXIDASE 1* (*SlRBOH1*) and enhanced H_2_O_2_ accumulation in the vascular cells of roots, stems, leaf petioles, and the leaf apoplast. Plants grown with higher CO_2_ levels showed improved salt tolerance, together with decreased leaf transpiration rates and lower sodium concentrations in the xylem sap, vascular tissues, and leaves. Silencing *SlRBOH1* abolished high CO_2_ -induced salt tolerance and increased leaf transpiration rates, as well as enhancing Na^+^ accumulation in the plants. The higher atmospheric CO_2_ level increased the abundance of a subset of transcripts involved in Na^+^ homeostasis in the controls but not in the *SlRBOH1*-silenced plants. It is concluded that high atmospheric CO_2_ concentrations increase salt stress tolerance in an apoplastic H_2_O_2_ dependent manner, by suppressing transpiration and hence Na^+^ delivery from the roots to the shoots, leading to decreased leaf Na^+^ accumulation.

## Highlight

High CO_2_ concentrations can counteract the negative impact of salt stress in an apoplastic H_2_O_2_-dependent manner by regulating stomatal movement and Na^+^ delivery from the xylem to leaf cells.

## Introduction

Crop productivity and food security are threatened by global climate change factors, such as projected temperature increases of more than 3.5 °C (Warren *et al.*, 2011; Meehl *et al.*, 2012). Moreover, atmospheric CO_2_ levels are likely to double by the end of this century (Solomon *et al.*, 2007). Crop yields are already limited in some areas by soil salinization, due in part to rising sea levels and agricultural practices such as irrigation and fertilization. Moreover, greenhouse crops, which are often grown with elevated levels of CO_2_, can experience salinity because of frequent irrigation and fertilization, which are common practices in greenhouse agriculture.

High atmospheric CO_2_ levels are likely to have a profound effect on oxidative signalling in plants, particularly because of the suppression of photorespiration (Munne-Bosch *et al.*, 2013). Atmospheric CO_2_ enrichment increases photosynthetic efficiencies, at least in the short term, leading to increased growth and biomass production (Foyer *et al.*, 2012). In contrast, salt stress decreases crop yields. Moreover, soil salinity is an important factor leading to the continuous loss of arable land (Shabala and Cuin, 2008). While many studies have focused either on plant responses to atmospheric CO_2_ enrichment or salt stress, there is a dearth of literature on plant responses to the combined effects of salinity and high CO_2_. High CO_2_ is known to induce salt tolerance (Yu *et al.*, 2015) but the molecular and metabolic mechanisms that underpin this response are poorly understood.

High salt concentrations have a severe impact on plant growth and metabolism, decreasing water uptake and inhibiting key metabolic processes such as photosynthesis (Flowers and Yeo, 1995; Mäser *et al.*, 2002; Deinlein *et al.*, 2014). High soil salinity enhances the production of reactive oxygen species (ROS), a process that is accompanied by increased membrane lipid peroxidation (Mittler, 2002; Miller *et al.*, 2010). Recent studies suggest that ROS are involved in the regulation salt tolerance (Bose *et al.*, 2013; Schmidt *et al.*, 2013). For example, *Arabidopsis thaliana* knockout mutants lacking the respiratory burst oxidase (Atrboh) F or D proteins, which are NADPH oxidases that catalyse the production of ROS in the apoplast, show increased salt sensitivity and altered Na^+^/K^+^ homeostasis (Ma *et al.*, 2012; Jiang *et al.*, 2012, 2013).

Many plants have evolved protective mechanisms to cope with salinity and minimize salt toxicity (Zhu, 2002; Munns and Tester, 2008). For example, in *Arabidopsis* the salt overly sensitive (SOS) signalling pathway is considered to mediate salt stress responses that regulate ion homeostasis (Ji *et al.*, 2013). The SOS1 transporter functions in long-distance transport of Na^+^ through the xylem from roots to shoots during salt stress (Shi *et al.*, 2002). The *SOS2* gene encodes a putative serine-threonine type protein kinase that is required for intracellular Na^+^ and K^+^ homeostasis (Liu *et al.*, 2000). Moreover, *SOS3* encodes a myristoylated calcium-binding protein that functions as a primary calcium (Ca^2+^) sensor, perceiving increases in cytosolic Ca^2+^ that are triggered by excess Na^+^ (Liu and Zhu, 1998; Halfter *et al.*, 2000; Liu *et al.*, 2000). As a result of SOS3-SOS2 interactions, SOS2 is recruited to the plasma membrane, a process that leads to the downstream activation of SOS1 and the extrusion of excess Na^+^ from the cytosol (Qiu *et al.*, 2002; Quintero *et al.*, 2011). In addition to SOS, other components such as Na^+^/H^+^ exchanger (NHX), high-affinity K^+^ transporter (HKT) transporters and mitogen-activated protein kinase (MAPK) are also important in salt stress tolerance and in root-to-shoot Na^+^ partitioning (Horie *et al.*, 2009; Bassil and Blumwald, 2014; Li *et al.*, 2014).

High atmospheric CO_2_ levels increase the photosynthesis/photorespiration ratio (Foyer *et al.*, 2012). The enhanced photosynthesis rates triggered by high CO_2_ levels are accompanied by decreased water loss through transpiration due to partial stomatal closure (Drake *et al.*, 1997; Robredo *et al.*, 2007). Elevated atmospheric CO_2_ levels also lead to the activation of carbonic anhydrase (CA) proteins (Hu *et al.*, 2010), a process that coincides with the activation of the open stomata 1 (OST1) protein kinase and the SLAC1 (slow anion channel 1) anion channel, which are involved in the regulation of stomatal movement (Tian *et al.*, 2015).

In addition to increasing photosynthetic CO_2_ assimilation rates, growth with high atmospheric CO_2_ levels can mitigate against the negative impacts of abiotic stresses (Ameye *et al.*, 2012; Bauweraerts *et al.*, 2013; Zinta *et al.*, 2014). For example, growth under high CO_2_ led to enhanced tolerance to salinity, Fe deficiency and increased resistance to (hemi) biotrophic microbes such as tobacco mosaic virus and *Pseudomonas syringae* in tomato (*Solanum lycopersicum*; Jin *et al.*, 2009; Takagi *et al.*, 2009; Del Amor, 2013; Li *et al.*, 2015). In contrast, growth under elevated CO_2_ enhanced susceptibility to the necrotrophic pathogen, *Botrytis cinerea* (Zhang *et al.*, 2015). Atmospheric CO_2_ enrichment enhanced photosynthesis rates and increased the growth of several plant species under saline conditions (Bowman and Strain, 1987; Robredo *et al.*, 2007; Del Amor, 2013). High CO_2_-induced salt tolerance is associated with reduced oxidative stress and transpiration rates, and with improved cellular hydration, intracellular Na^+^/K^+^ homeostasis and water use efficiency (Bowman and Strain., 1987; Maggio *et al.*, 2002; Yu *et al.*, 2015). However, the mechanisms by which high atmospheric CO_2_ levels suppress transpiration and hence decrease the delivery of Na^+^ from roots to shoots remain to be characterized.

The tomato respiratory burst oxidase RBOH1 gene, *SlRBOH1*, is a homologue of *Arabidopsis RBOHF*. It has the highest transcript abundance within the RBOH family (Zhou *et al.*, 2014*b*). *SlRBOH1* is involved in the regulation of tolerance to oxidative stress and to high temperature stress. It also plays a key role in the regulation of stomatal movements mediated by the phytohormones, abscisic acid (ABA) and brassinosteroid (BR) (Nie *et al.*, 2013; Xia *et al.*, 2014; Zhou *et al.*, 2014*a*). Apoplastic ROS are involved in the regulation of stomatal movement and Na^+^/K^+^ homeostasis (Jiang *et al.*, 2012). In order to test the hypothesis that elevated CO_2_-induced salt tolerance is linked to apoplastic H_2_O_2_ accumulation, the impact of high atmospheric CO_2_ concentrations on the responses to tomato plants to salt stress was evaluated, with a particular focus on the role of *SlRBOH1*.

## Materials and methods

### Plant materials

Tomato (*S. lycopersicum* L cv. Ailsa Craig) seeds were sown in perlite and kept at 28 °C for 2 weeks. Seedlings were then transferred to plastic tanks (20 cm×30 cm×15cm, 4 seedlings per tank) filled with Hoagland nutrient solution in growth chambers. The growth conditions used for subsequent growth of the plants were as follows: a 14/10h (day/night) photoperiod, a temperature regime of 25/20 °C (day/night), a photosynthetic photo flux density (PPFD) of 600 µmol m^–2^ s^–1^ and a relative humidity of 85%. Plants at the 4-leaf stage were used for Experiment I.

### Virus-induced gene silencing (VIGS) of *RBOH1*

The tobacco rattle virus (TRV) VIGS constructs used for silencing of the *SlRBOH1* gene were generated by cloning a 311-bp RBOH1 cDNA fragment, which was amplified by PCR using the forward primer (5′-ATACGCGAGCTCAAGAATGGGGTTGATATTGT-3′) and reverse primer (5′-CGGAATTCAGACCCTCAACACT CAACCC-3′). The amplified fragment was digested with the restriction endonucleases, *Sac*I and *Xho*I, and ligated into the same sites of the pTRV2 vector. The resulting plasmid was transformed into *Agrobacterium tumefaciens* strain GV3101, and VIGS was performed by infiltration of 15-d-old wild-type seedlings with a mix of *A. tumefaciens* strains harbouring pTRV1- or pTRV2 (Ekengren *et al.*, 2003). Plants were then kept at 22 °C under a 14-h photoperiod for 30 d before they were used for Experiment II (Kandoth *et al.*, 2007).

### Salt and atmospheric CO_2_ enrichment treatments

Both experiments involving salt and atmospheric CO_2_ treatments were carried out in CO_2_-controlled growth chambers (ConvironE15; Conviron, Manitoba, Canada). Plants were kept at 25/20 °C, with a 14-h photoperiod under 600 μmol m^–2^ s^–1^ PPFD and 85% humidity conditions. In Experiment I, tomato plants at the 4-leaf stage were exposed to ambient CO_2_ concentrations (380 μmol mol^–1^), ambient CO_2_ concentration with 100mM NaCl in the nutrient solution, elevated CO_2_ (760 μmol mol^–1^), and elevated CO_2_ with 100mM NaCl in the nutrient solution. In Experiment II, pTRV and pTRV-*RBOH1* plants at the 5-leaf stage were exposed to two levels of CO_2_ (380 and 760 μmol mol^–1^) and a nutrient solution with or without NaCl (100mM) for 11 d, giving a total of eight treatments for Experiment II. The nutrient solution was replaced every 3 d during the experiments and all measurements were performed with at least four replicates, with 20 plants per replicate. Leaf or root samples were frozen in liquid nitrogen and stored at –80 °C until used for the biochemical assays and gene transcript analyses. During the experiments, plants were harvested and oven-dried at 80 °C for 3 d before determination of dry weight and measurement of Na^+^ and K^+^ contents.

### Physiological and biochemical measurements

The CO_2_ assimilation rates (Pn), transpiration rates (Tr), and stomatal conductance (Gs) of the plants were determined with an infrared gas analyzer-based portable photosynthesis system (LI-6400; LI-COR, Lincoln, NE, USA). The air temperature, relative humidity, and PPFD were maintained at 25 °C, 85% and 1000 μmol m^–2^ s^–1^, respectively, with variable CO_2_ concentrations. The maximum quantum yield of photosystem II (PSII) (*F*v/*F*m) was measured using an imaging-PAM chlorophyll fluorimeter equipped with a computer-operated PAM-control unit (IMAG-MAXI; Heinz Walz, Effeltrich, Germany) as previously described (Zhou *et al.*, 2014*a*). The seedlings were kept in the dark for at least 30min before the measurements were taken. *F*v/*F*m values were calculated as *F*v/*F*m = (*F*m−*F*o)/*F*m, where *F*o is the minimal chlorophyll fluorescence measured during the weak measuring pulses and *F*m is the maximum fluorescence measured by a 0.8 s pulse light at 4000 μmol mol^−2^ s^−1^. *F*v/*F*m values were determined using the whole leaf.

Relative electrolyte leakage was measured in the leaves as previously described (Cao *et al.*, 2007) using a conductivity detector (FE30K, Mettler-Toledo Instruments Co., Ltd., Switzerland). Briefly, leaf segments (0.3g) were vacuum-infiltrated in 20ml deionized water for 20min and kept in the water for 2h, and the conductivity (C1) of the resulting solutions were then determined. The leaf segments were then boiled for 15min, cooled to room temperature, and the conductivity (C2) of the resulting solutions were determined. The C1:C2 ratios (C1/C2×100%) were calculated and used as a measure of the relative electrolyte leakage. The level of lipid peroxidation in leaves was assessed by measuring the malonyldialdehyde (MDA) content using 2-thiobarbituric acid as described by Hodges *et al.* (1999). Leaf water potential of intact excised leaves was measured using a Dew point Potential Meter (WP4; Decagon Device, Pullman, USA). Plant cell death was detected by Trypan Blue staining as previously described (Bai *et al.*, 2012). Briefly, leaves were boiled for 5min in a 1:1 mixture of ethanol and staining solution (10ml lactic acid, 10ml glycerol, 10ml phenol, and 10mg Trypan Blue dissolved in 10ml distilled water) for staining. The leaves were then de-stained with chloral hydrate (2.5g ml^−1^ distilled water), changing the solution every 12h until the leaves were transparent. The stomatal apertures were measured as previously described (Xia *et al.*, 2014) by peeling off the abaxial epidermis with forceps and floating it on a buffer containing 30mM KCl and 10mM 2-(*N*-morpholino)-ethanesulfonic acid, at a temperature of 25 °C. All images were captured using a light microscope equipped with a digital camera (Leica Microsystems, Wetzlar, Germany). NADPH oxidase activity was determined in isolated plasma membrane vesicles as previously described (Zhou *et al.*, 2014*b*).

### Visualization of cellular H_2_O_2_ and Na^+^ accumulation

Hydrogen peroxide (H_2_O_2_) production in tissues was monitored using 2,7-dichlorofluorescein diacetate (H_2_DCF-DA), as previously described (Pei *et al.*, 2000; Milling *et al.*, 2011; Xia *et al.*, 2011) with minor modifcations. Detached roots were washed with deionized water and incubated 15min with 25 μM H_2_DCF-DA in 200mM phosphate buffer (pH 7.4) and then washed five times with the same buffer without the dye. As negative controls, the root segments were incubated with 1mM ascorbate (ASC) or 100U ml^–1^ catalase (H_2_O_2_ scavenger) for 30min. The stem and petiole were excised using a scalpel and incubated for 15min in 200mM phosphate buffer (pH 7.4), then incubated for 15min with 25 μM H_2_DCF-DA in 200mM phosphate buffer (pH 7.4), and washed as described above. Fluorescence was observed using a Leica DM4000B microscope and images were captured using a Leica DFC425C camera and the Leica application suite V3.8 software (Leica Microsystems, Germany).

H_2_O_2_ in the leaf apoplast was visualized using cytochemical CeCl_3_ staining. Leaf fragments (3mm^2^) were excised from inoculated leaf panels and infiltrated with freshly prepared 5mM CeCl_3_ in 50mM Mops at pH 7.2 for 1h at 28 °C, and then fixed and embedded according to Bestwick *et al.* (1997). Leaf sections were examined using a transmission electron microscope (H7650, Hitachi, Tokyo, Japan) at an accelerating voltage of 75kV, to reveal any electron-dense CeCl_3_ deposits that are formed in the presence of H_2_O_2_. H_2_O_2_ was extracted and analysed as previously described (Willekens *et al.*, 1997), in which plant material was first ground in liquid nitrogen and HClO_4_. After thawing and centrifugation, the supernatants were adjusted to pH 6.0 with KOH and passed through columns to remove lipid peroxides. H_2_O_2_ was then quantified using a glutathione peroxidase/horseradish peroxidase assay system.

The detection of Na^+^ in roots was carried out as described by Oh *et al.* (2009). Tomato plants were treated with 100mM NaCl in Hoagland nutrition solution for 3 d, after which, whole root systems were incubated for 8h in a Petri dish with the same media supplemented with 20 μM CoroNa Green-AM (Invitrogen). The tissue was washed with 200mM phosphate buffer (pH 7.4) five times to remove excess dye and observed using a Leica DM4000B microscope (Leica Microsystems, Wetzlar, Germany). Images were captured using a Leica DFC425C camera and the Leica application suite V3.8 software.

### Determination of ion content in tissues and xylem sap

The ion content in tissues and xylem sap was measured as previously described (Jiang *et al.*, 2012). Dried root, stem, and leaf material (0.3g) was digested in 5ml concentrated HNO_3_ (69%, v/v) for at least 12h before extraction. The solution was then filtered, using quantitative filter paper and diluted with deionized water. Concentrations of Na^+^ and K^+^ in the diluted samples were determined in an air–acetylene flame using an atomic absorption spectrometer (A6300; Shimadzu, Kyoto, Japan). During the experiment, the xylem sap exuding from the cut stem surfaces of 12 replicate de-topped plants was collected and pooled, then diluted with deionized water for ion content measurement, prior to quantification by atomic absorption spectrophotometry.

### RNA extraction and quantitative RT-PCR analysis

RNA was extracted from leaves and roots using the RNAprep pure Plant Kit (Tiangen biotech Co., Ltd. Beijing, China) and quantified using a NanoDrop ND-2000 Spectrophotometer (Thermo Scientific, Waltham, MA, USA), before quality assessment with a gel cartridge on a Bio-Rad platform (Bio-Rad, Hercules, CA, USA). RNA samples were used only if the ribosomal bands showed no degradation, and the 260/280 and 260/230 absorbance ratios were between 1.8 and 2.1. Total RNA (1 μg) was reverse transcribed using a ReverTra Ace quantitative (qPCR) RT Kit (Toyobo, Osaka, Japan), following the manufacturer’s instructions. qRT-PCR was performed using the LightCycler 480 Real-Time PCR System (Roche Diagnostics, Germany). Each reaction (20 μl) consisted of 10 μl SYBR Green PCR Master Mix (Takara, Chiga, Japan), 8.6 μl sterile water, 1 μl cDNA, and 0.2 μl forward and reserve primers (10 μM). PCR was performed with 40 cycles of 30 s at 95 °C, 30 s at 58 °C, and 1min at 72 °C. Gene-specific primers are listed in Supplementary Table S1 (available at *JXB* online) and Actin2 was used as the reference gene. The relative gene expression was calculated according to Livak and Schmittgen (2001).

### Statistical analysis

The experimental design was a completely randomized block design with four replicates. Each replicate contained 16 plants and at least four independent replicates were used for each determination. The data were subjected to analysis of variance, and the means were compared using Tukey’s test at the 5% level.

## Results

### CO_2_ enrichment attenuates salt-induced growth reduction, cellular membrane peroxidation, and Na accumulation but increases the levels of *RBOH1* transcripts

**Fig. 1.**
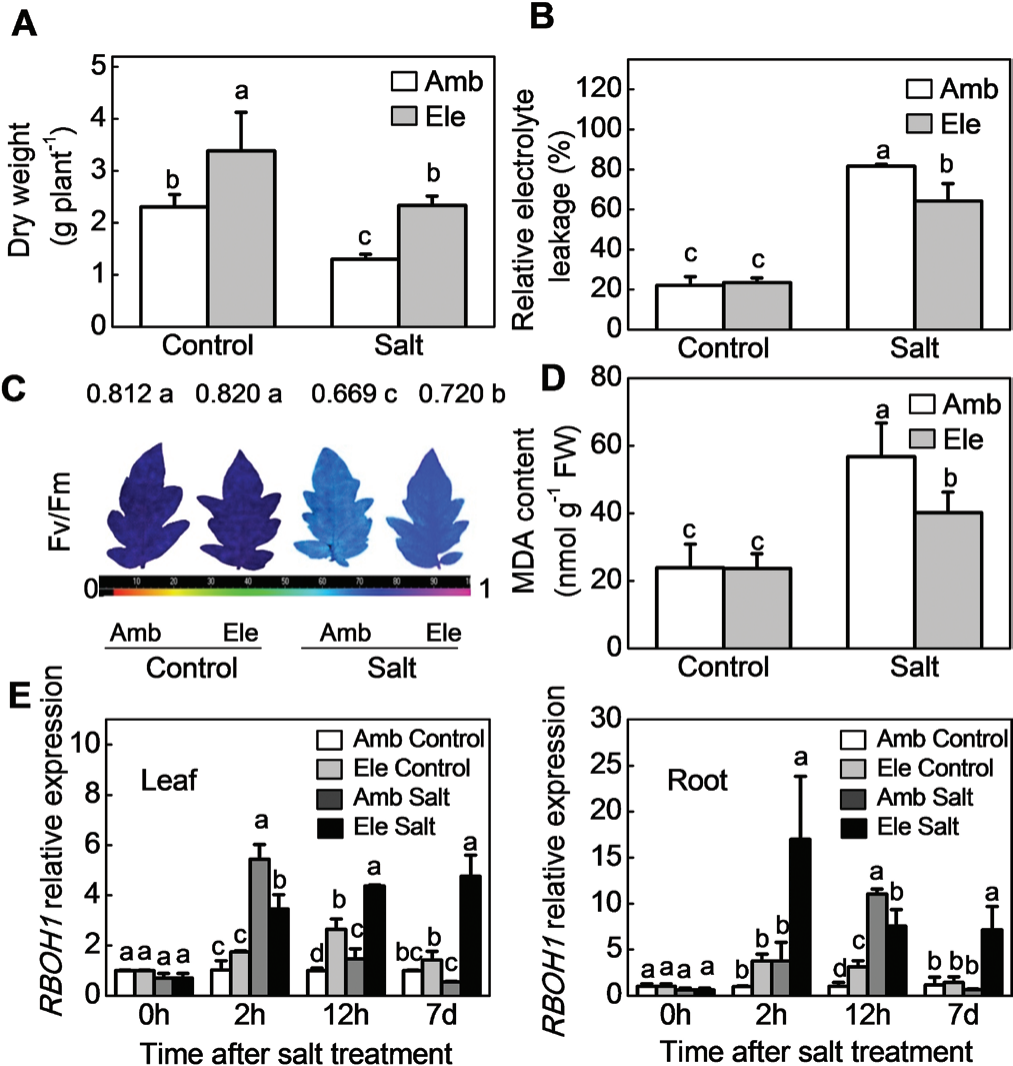
Salinity tolerance and *RBOH1* transcript levels in tomato plants grown under elevated (Ele: 760 µmol mol^–1^) or ambient (Amb: 380 µmol mol^–1^) CO_2_ conditions. (A) Dry weight. (B) Relative electrolyte leakage. (C) Images of the maximum photochemical efficiency of PSII (*Fv/Fm*). The false colour code depicted at the bottom of the image ranges from 0 (black) to 1.0 (purple). The value at the top of the image indicates actual value. (D) MDA content in leaves. (E) *RBOH1* transcript levels in leaves (left) and roots (right). Samples for dry weight, relative electrolyte leakage, and MDA analyses were harvested 11 d after salt treatment, while *Fv/Fm* were measured 3 d after salt treatment. The data values are the means ± standard deviation (SD) of four replicates. Means denoted by the same letter did not differ significantly according to Tukey’s test (*P*<0.05). For Fig. 1E, significant differences between treatments within the same time are indicated by different letters.

Biomass accumulation was significantly higher (~47%) in tomato plants grown with CO_2_ enrichment than under ambient CO_2_ conditions (Fig. 1A). While biomass was reduced by growth in the presence of salt, the salt-induced reduction in biomass was significantly lower in plants grown with CO_2_ enrichment than under ambient CO_2_ conditions (Fig. 1A). High CO_2_-grown plants had lower levels of electrolyte leakage and of MDA, which is an end product of lipid peroxidation. They also showed lower salt-induced decreases in photosynthetic CO_2_ assimilation (Pn) and in the maximum quantum yield of photosystem II (PSII) (*Fv/Fm*, Fig. 1B–D, Supplementary Fig. S1 available at *JXB* online). However, stomatal conductance (Gs) values and transpiration rates (Tr) were lower in plants grown with CO_2_ enrichment than under ambient CO_2_ conditions, in the presence or the absence of salt stress (Supplementary Fig. S1).

**Fig. 2.**
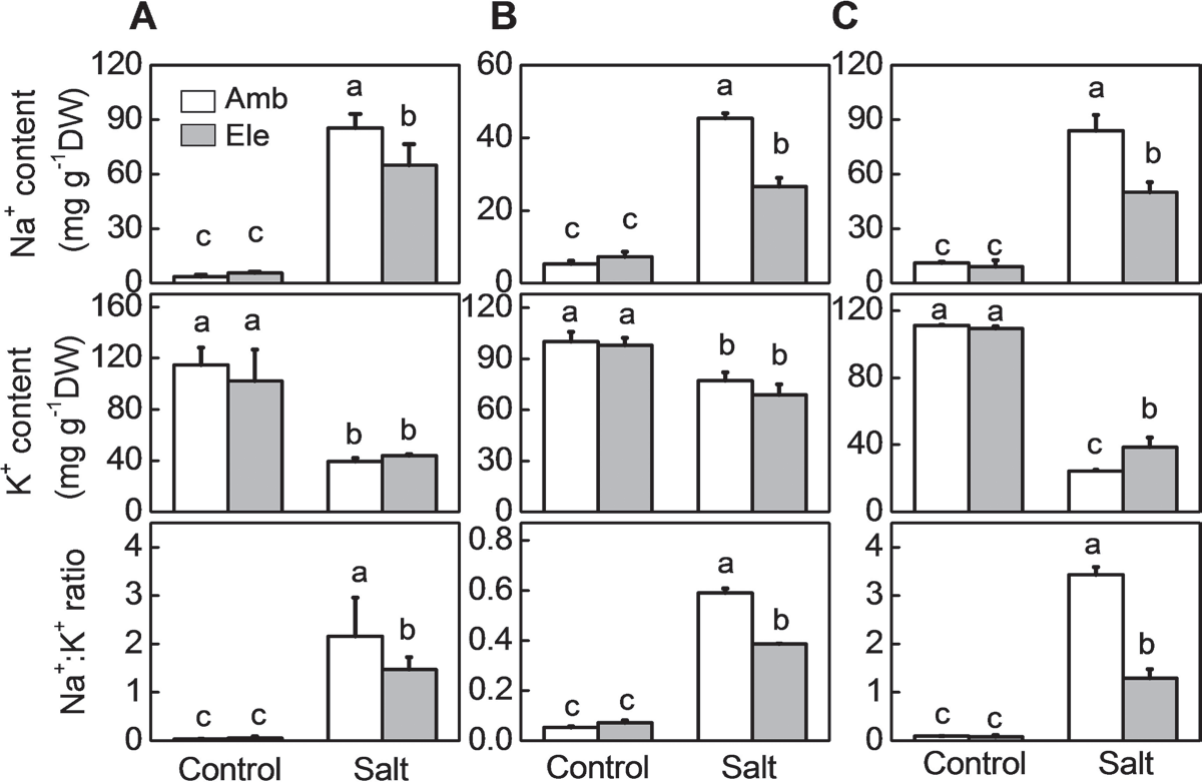
Effects of NaCl treatments on accumulation of Na and K and the Na^+^:K^+^ ratio in tomato plants grown under elevated (Ele: 760 µmol mol^–1^) or ambient (Amb: 380 µmol mol^–1^) CO_2_ conditions. (A) Leaves. (B) Stems. (C) Roots. Samples were taken 11 d after salt treatment. The data are means ± SD of four replicates.

Exposure to either salt stress or elevated CO_2_ increased the levels of *RBOH1* transcripts in leaves and roots (Fig. 1E). However, the effects of high salt on *RBOH1* transcripts were greatest in plants grown under high CO_2_. The levels of H_2_O_2_ were measured both qualitatively, using H_2_DCF-DA as a fluorescence probe, and quantitatively, using a spectrophotometric assay method. Both of these methods showed increased H_2_O_2_ accumulation in the roots and petioles of salt-treated plants that were grown under high CO_2_ compared with ambient CO_2_ conditions (Supplementary Figs S2A, B and S3). Moreover, the salt-induced fluorescence signal was greatly decreased in the presence of ascorbate or catalase, confirming the specificity of H_2_DCF-DA for H_2_O_2_ detection (Supplementary Fig. S2C). Crucially, growth with CO_2_ enrichment significantly decreased Na^+^ accumulation in roots, stems, and leaves but it increased K^+^ accumulation in roots, leading to a reduction in the Na^+^:K^+^ ratio in these organs (Fig. 2).

### Silencing of *RBOH1* compromises the salt tolerance conferred by elevated CO_2_ levels

**Fig. 3.**
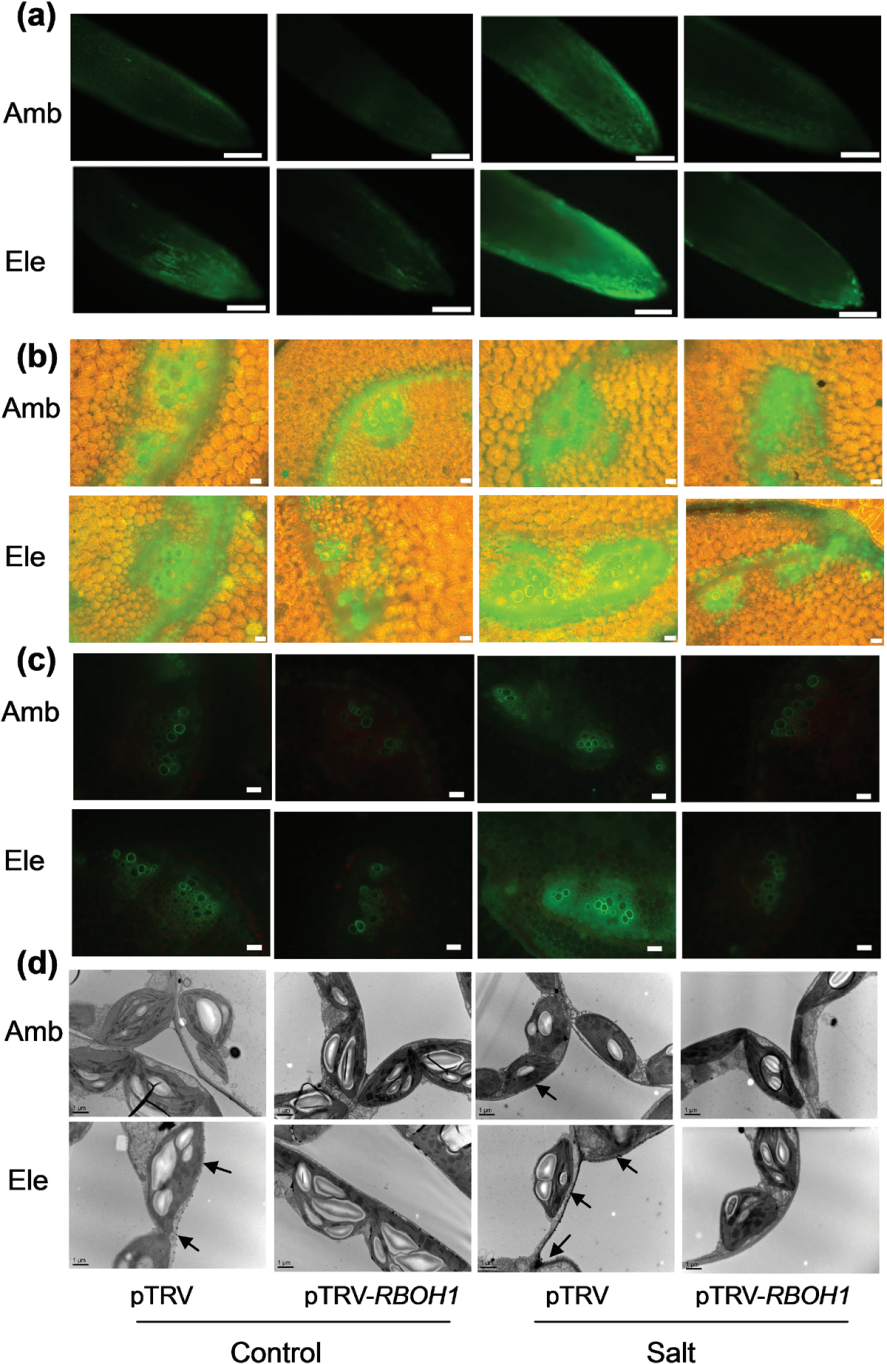
Effects of NaCl treatments on H_2_O_2_ accumulation in pTRV empty vector control and *RBOH1* silenced tomato plants grown under elevated (Ele: 760 µmol mol^–1^) or ambient (Amb: 380 µmol mol^–1^) CO_2_ conditions. (A) H_2_O_2_ accumulation in roots. Scale bars = 200 μm. (B) H_2_O_2_ accumulation in stems. Scale bars = 50 μm. (C) H_2_O_2_ accumulation in petioles. Scale bars = 200 μm. (D) Cytochemical localization of H_2_O_2_ accumulation in leaf mesophyll cells as visualized by CeCl_3_ staining and TEM. Samples were harvested 3 d after salt treatment. The root and petiole images were captured using fluorescence contrast method, and the stem images using fluorescence-phase contrast method. The arrows in (D) indicate CeCl_3_ precipitates. Scale bars = 1 μm.

*RBOH1* transcript levels were reduced by 53.6% in tomato plants subjected to virus-induced gene silencing (pTRV-*RBOH1*) compared with empty vector (pTRV) controls (Supplementary Fig. S4). However, no significant changes were found in the transcript levels of other RBOHs in the pTRV-*RBOH1* plants. NADPH oxidase activity was decreased by 38.1 % in the leaves of the pTRV-*RBOH1* plants compared with the pTRV controls. Both CO_2_ enrichment and high salt conditions induced H_2_O_2_ accumulation in roots, stems, leaf petioles, and leaves in the pTRV plants (Fig. 3). Salt- and CO_2_-induced H_2_O_2_ accumulation was primarily localized within the parenchyma cells of the vascular tissue in the pericycle in the leaf petioles (Fig. 3C). Moreover, H_2_O_2_ was predominately localized in the apoplast/cell wall compartment of the salt-treated leaf cells (Fig. 3D). Salt and high CO_2_-dependent H_2_O_2_ accumulation was much lower in the pTRV-*RBOH1* plants than the pTRV controls (Fig. 3; Supplementary Fig. S5).

**Fig. 4.**
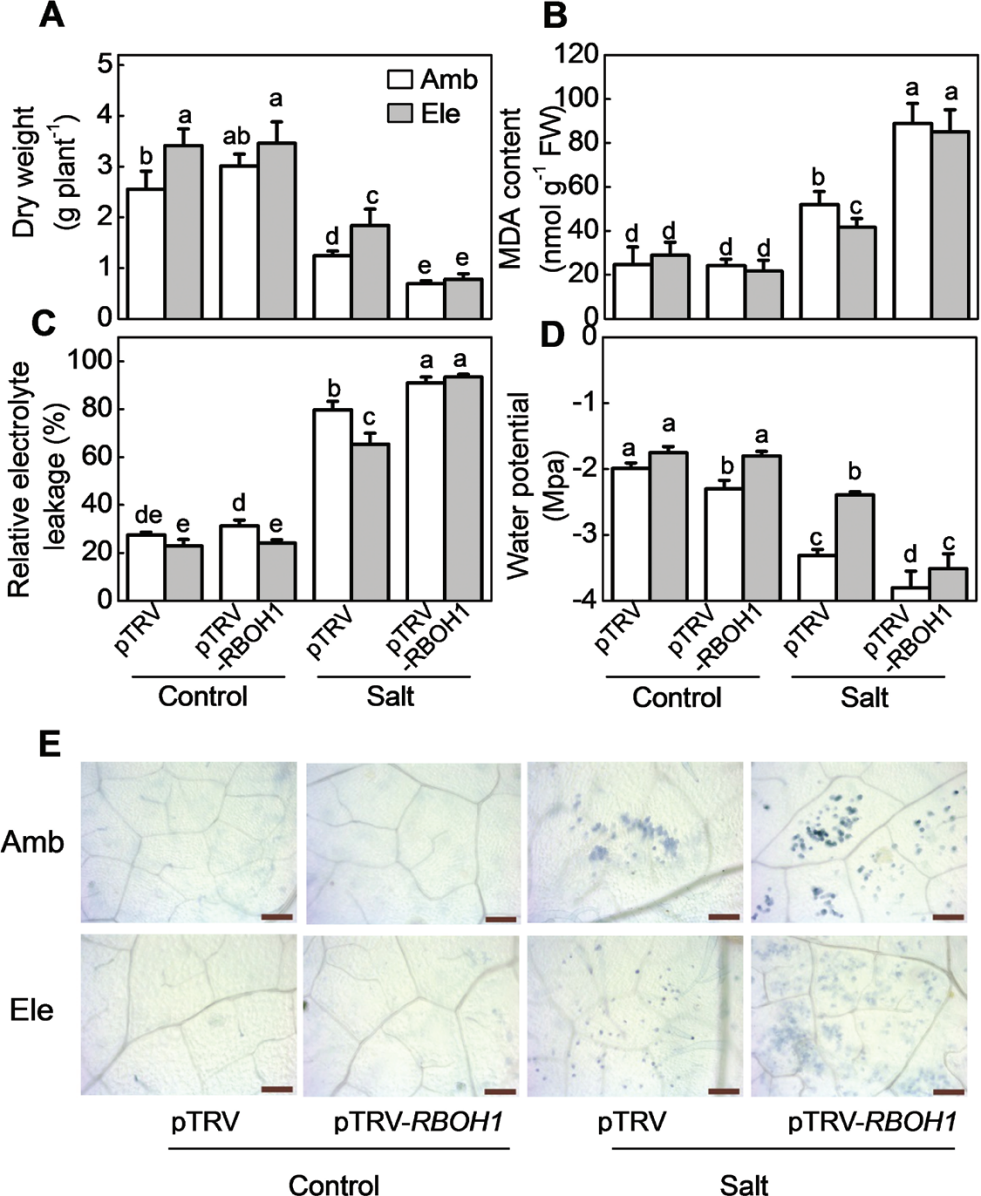
Salinity tolerance in pTRV empty vector control and *RBOH1* silenced tomato plants grown under elevated (Ele: 760 µmol mol^–1^) or ambient (Amb: 380 µmol mol^–1^) CO_2_ conditions. (A) Dry weight. (B) MDA content in leaves. (C) Relative electrolyte leakage in leaves. (D) Water potential. (E) Leaves stained with Trypan Blue. Scale bars = 10 μm. Dry weight, MDA, relative electrolyte leakage, and cell death were determined with plant material harvested 11 d after salt treatment, and water potential was measured 3 d after salt treatment. The data values are the means ± SD of four replicates. Bars denoted by the same letter did not differ significantly according to Tukey’s test (*P*<0.05).

While the growth of the pTRV-*RBOH1* plants was not significantly different from that of the pTRV controls in the absence of salt, the negative effects of high salt on biomass accumulation, electrolyte leakage, lipid peroxidation, and cell viability were increased in pTRV-*RBOH1* plants relative to pTRV controls (Fig. 4). CO_2_ enrichment attenuated the salt-induced decreases in biomass accumulation, tissue water potentials as well as reducing the salt-induced increases in electrolyte leakage and MDA contents in the pTRV controls, but not in the pTRV-*RBOH1* plants (Fig. 4).

### *RBOH1*-derived H_2_O_2_ is associated with Na^+^ transport from roots to leaves

**Fig. 5.**
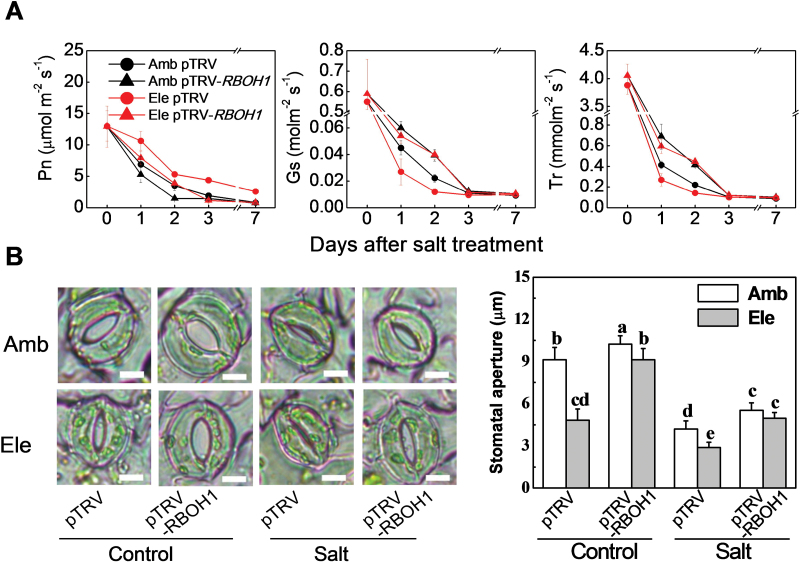
Effects of NaCl treatments on gas exchange and stomatal movement in pTRV empty vector control and *RBOH1* silenced tomato plants grown under elevated (Ele: 760 µmol mol^–1^) or ambient (Amb: 380 µmol mol^–1^) CO_2_ conditions. (A) CO_2_ assimilation rate (Pn), stomatal conductance (Gs), and transpiration (Tr). (B) Stomatal movement and stomatal aperture. The data are means ± SD of four replicates except for stomatal aperture which are the average means of three biological replicates, and each replicate is the average value of stomata in a field of microscope (with about 25 stomata) under each treatment. Scale bars = 10 µm.

Photosynthesis rates (Pn) were similar in the pTRV-*RBOH1* plants and pTRV controls but the stomatal conductance (Gs) values, stomatal aperture sizes, and transpiration rates (Tr) were slightly higher in the pTRV-*RBOH1* leaves than the pTRV controls (Fig. 5). The salt-induced decreases in Gs and Tr were significantly higher in plants grown under high CO_2_ than under ambient CO_2_ conditions (Fig. 5). The *RBOH1* silenced tomato plants had consistently higher Gs, Tr, and stomatal aperture values (Fig. 5). High atmospheric CO_2_ had little effect on the Gs and Tr values (Fig. 5).

**Fig. 6.**
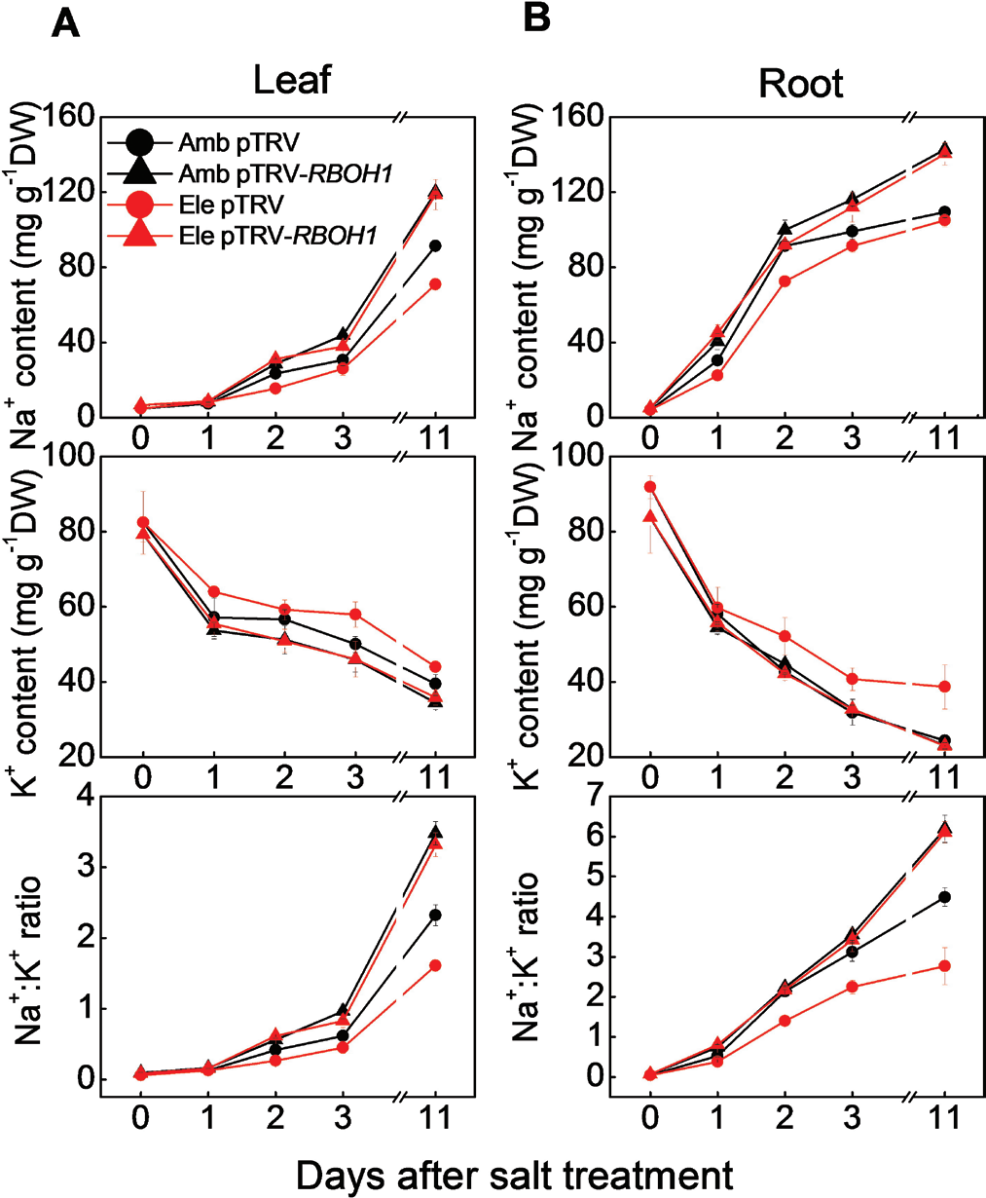
Effects of NaCl treatments on accumulation of Na^+^ and K^+^, and the Na^+^:K^+^ ratio in pTRV empty vector control and *RBOH1* silenced tomato plants grown under elevated (Ele: 760 µmol mol^–1^) or ambient (Amb: 380 µmol mol^–1^) CO_2_ conditions. (A), Na^+^, K^+^ content, and the Na^+^:K^+^ ratio in leaves. (B), Na^+^ and K^+^ content, and the Na^+^:K^+^ ratio in roots. The data are means ± SD of four replicates.

The levels of Na^+^ were higher in the pTRV-*RBOH1* leaves than the pTRV controls under salt stress. In contrast, in the presence of salt, the leaf K^+^ levels were lower in the pTRV-*RBOH1* leaves than the pTRV controls (Fig. 6). When the pTRV controls were grown with high CO_2_, leaf Na^+^ accumulation decreased and leaf K^+^ levels increased. Hence, the Na^+^:K^+^ ratios were decreased in the salt-treated pTRV controls grown under high CO_2_ relative to plants grown under ambient CO_2_ conditions (Fig. 6). In contrast, the pTRV-*RBOH1* plants did not show an alleviation of salt-induced changes in Na^+^ and K^+^ accumulation in either leaves or roots when these plants were grown under elevated CO_2_ concentrations. Moreover, the Na^+^:K^+^ ratios of the salt-treated pTRV-*RBOH1* roots and shoots were similar under ambient or high CO_2_.

**Fig. 7.**
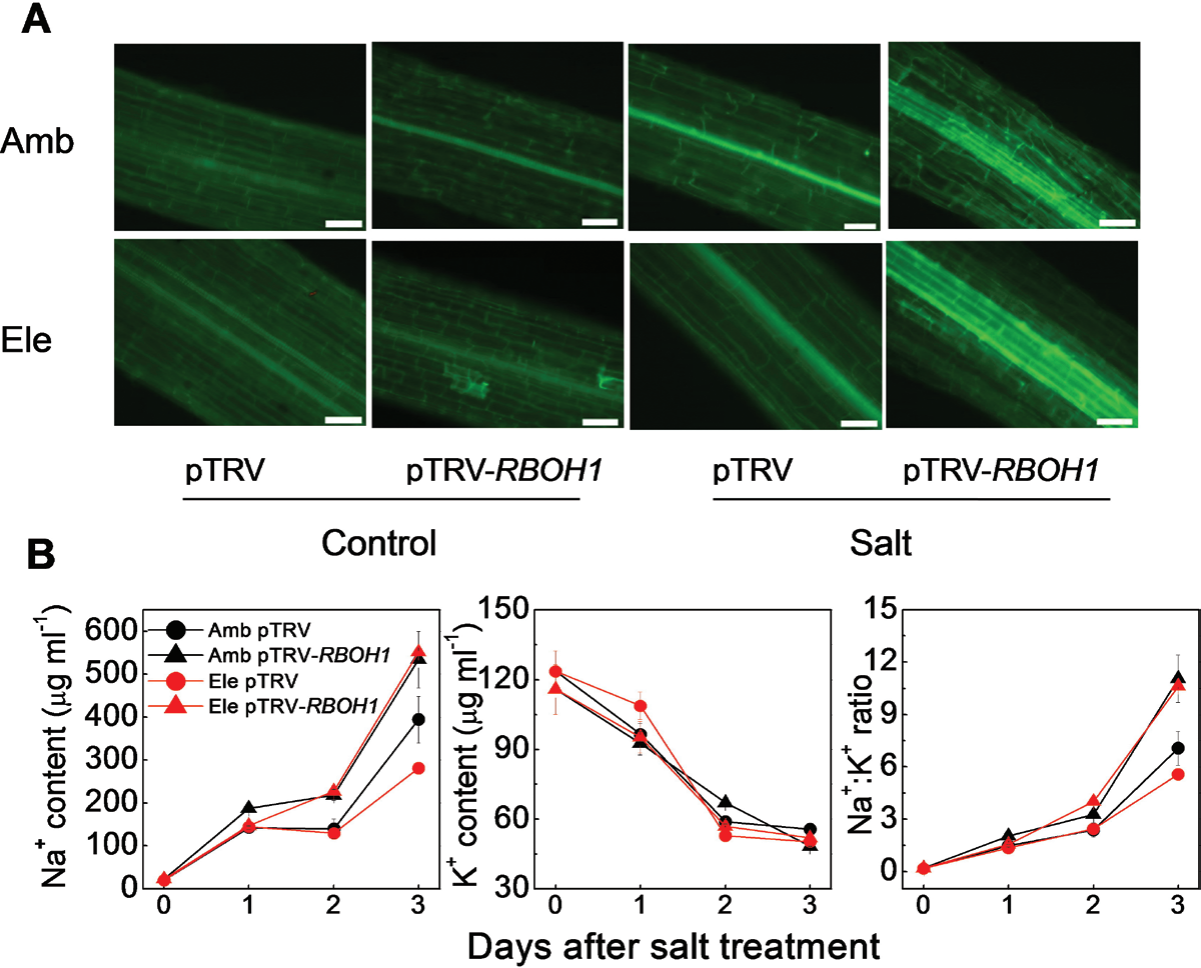
Effects of NaCl treatments on the ion location and content in the vascular tissue of pTRV empty vector control and *RBOH1* silenced tomato plants grown under elevated (Ele: 760 µmol mol^–1^) or ambient (Amb: 380 µmol mol^–1^) CO_2_ conditions. (A) Cellular localization of Na^+^ in roots. Confocal images of roots stained with CoroNa Green are shown. Samples were imaged 3 d after salt treatment. (B) Na^+^ and K^+^ content, and the Na^+^:K^+^ ratio in xylem sap.

Visualization of Na^+^ accumulation in roots was performed on plants exposed to high salt using CoroNa Green, a green-fluorescent indicator, whose emission intensity increases upon Na^+^ binding (Oh *et al.*, 2009). CoroNa-Green fluorescence was barely detectable in the roots of pTRV controls and pTRV-*RBOH1* plants grown under ambient CO_2_ in the absence of salt stress. In contrast, a strong fluorescent signal was observed in the pericycle and vascular cells of the roots of both pTRV controls and pTRV-*RBOH1* plants exposed to salt (Fig. 7A). When plants were grown under high CO_2_, fluorescence was only detected in one cell layer in the middle of the pTRV controls roots. In contrast, several cell layers in the high CO_2_-grown pTRV-*RBOH1* roots showed high fluorescence.

The xylem-sap Na^+^ concentration increased progressively over time in both pTRV-*RBOH1* plants and pTRV controls following the onset of salt treatment (Fig. 7B). However, the increase in xylem-sap Na^+^ concentrations were consistently higher in the pTRV-*RBOH1* plants than the pTRV controls (Fig. 7B). Exposure to high CO_2_ decreased xylem-sap Na^+^ concentrations in the pTRV control plants 3 d after the onset of the salt stress treatment but not in the pTRV-*RBOH1* plants. The pTRV controls showed the lowest xylem sap Na^+^:K^+^ ratios under high CO_2_ growth conditions. In contrast, the pTRV-*RBOH1* plants had the highest xylem sap Na^+^:K^+^ ratios under both ambient and high atmospheric CO_2_ levels.

### Apoplastic H_2_O_2_ plays a role in CO_2_-induced Na^+^ and K^+^ homeostasis

**Fig. 8.**
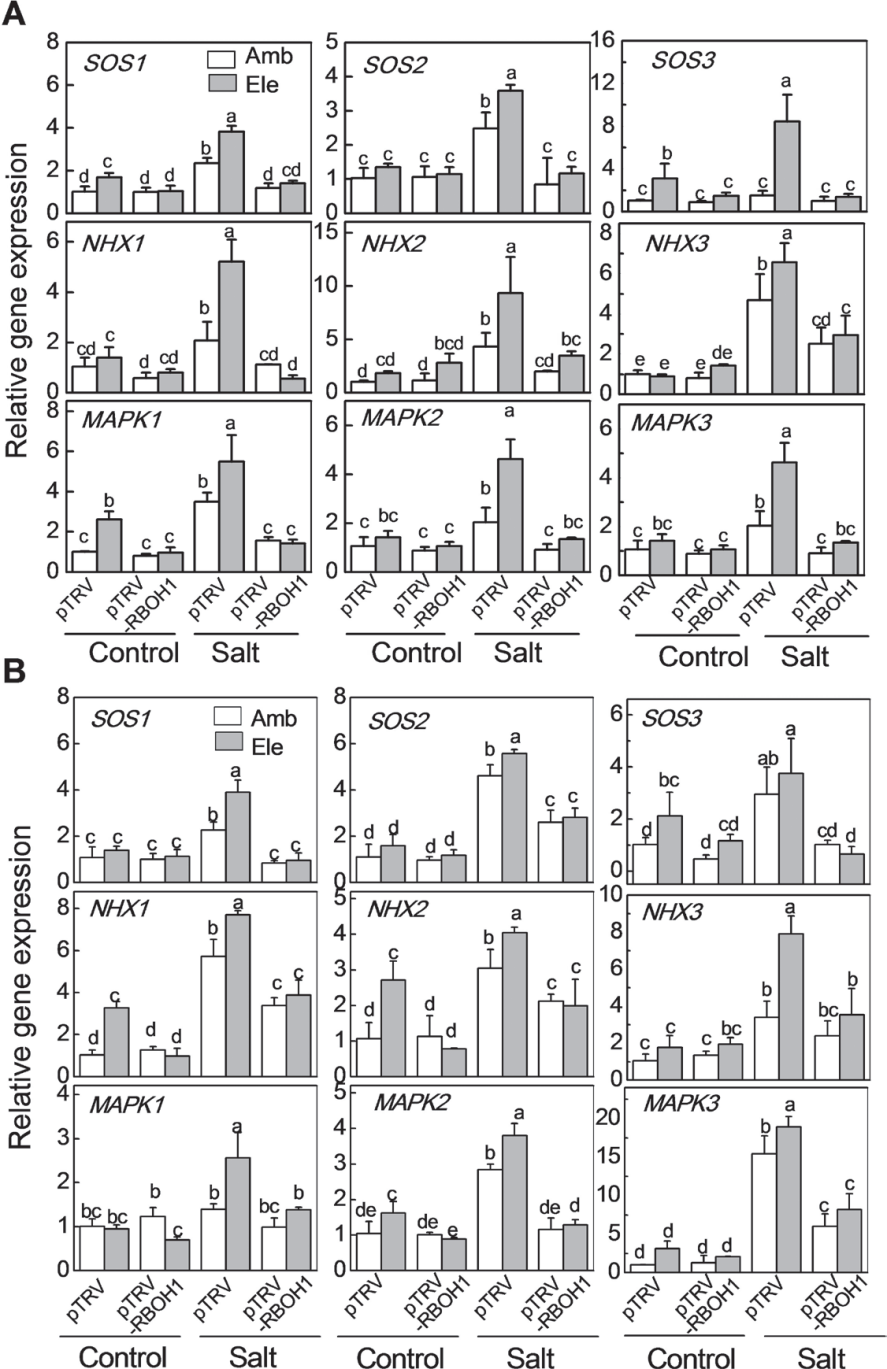
Effects of NaCl treatments on the transcript levels of genes involved in the SOS pathway, NHXs, and MAPKs in the pTRV empty vector control and *RBOH1* silenced tomato plants grown under elevated (Ele: 760 µmol mol^–1^) or ambient (Amb: 380 µmol mol^–1^) CO_2_ conditions. (A) Transcript levels in leaves. (B) Transcript levels in roots. Gene expression was evaluated 2 d after salt treatment. The data are means ± SD of four replicates. Bars denoted by the same letter did not differ significantly according to Tukey’s test (*P*<0.05).

When the pTRV-*RBOH1* plants were grown under ambient CO_2_ in the absence of salt, the levels of transcripts encoding salt response genes such as *SOS1*-*3* and *NHX1-3* (Ji *et al.*, 2013; Bassil and Blumwald, 2014) and *MAPK1-3* (Li *et al.*, 2014) were similar to the pTRV controls (Fig. 8). Moreover, the abundance of transcripts encoding salt response genes was similar in plants grown under ambient or high CO_2_. However, *SOS1*, *SOS3*, and *MAPK1* transcript levels in the leaves and *SOS3*, *NHX1*, *NHX2*, and *MAPK2* transcript levels in the roots, were significantly higher in plants grown under CO_2_ enrichment.

With the exception of *SOS3*, growth with high salt significantly increased the levels of transcripts encoding salt response genes in pTRV leaves and roots. In contrast salinity had negligible effects on the levels of most of measured transcripts, except for *NHX3* in the leaves, and *SOS2*, *NHX1*, *NHX2*, and *MAPK1* in the roots, which were significantly increased as a result of the salt treatment in pTRV-*RBOH1* plants. Crucially, growth under high CO_2_ had no effect on the levels of transcripts encoding the salt response genes in the pTRV-*RBOH1* plants.

## Discussion

Although plant responses to atmospheric CO_2_ enrichment and soil salinity are well characterized, relatively little is known about the interactions between these environmental stresses. In general, high CO_2_ levels promote plant growth due to increased photosynthesis, while high salinity inhibits growth by disrupting cellular Na^+^/K^+^ homeostasis (Zhu, 2003; Shabala and Cuin, 2008). However, salt stress has milder effects on the metabolism and physiology of plants grown under high CO_2_ conditions than those grown in air (Kanani *et al.*, 2010). The data presented here shows that growth under high CO_2_ alleviates the negative impacts of high NaCl on photosynthesis and biomass production (Figs 1 and 5; Supplementary Fig. S1). These findings agree with those of other studies concerning the relationships between elevated CO_2_ levels and high salinity in tomato and other plant species Takagi *et al.*, 2009; (Del Amor, 2013; Zaghdoud *et al.*, 2013; Pinero *et al.*, 2014; Yu *et al.*, 2015). High CO_2_ levels decreased leaf transpiration rates and stomatal conductance values, even in plants grown with high salt (Fig. 5A; Supplementary Fig. S1). The high CO_2_–dependent decreases in transpiration resulted in a decreased Na^+^ accumulation and lower Na^+^: K^+^ ratios in the tissues (Figs 2, 6, and 7B). These results suggest that the higher salt stress tolerance observed under elevated CO_2_ is largely dependent on the suppression of transpiration. This conclusion is supported by other studies showing that high CO_2_ alleviated the adverse effects of salinity by modulation of aquaporins leading to lower transpiration rates (Zaghdoud *et al.*, 2013).

The excessive accumulation of Na^+^ in leaves of plants grown under salt stress is dependent on both the transpiration rate and also the Na^+^ concentration in the transpiration stream. In turn, the transpiration rate is controlled by the degree of stomatal closure, which is related to the apoplastic production of H_2_O_2_ (Desikan *et al.*, 2005; Bright *et al.*, 2006; Desikan *et al.*, 2006). *RBOH1*-mediated H_2_O_2_ production not only plays a role in the control of stomatal movements but also in the acquisition of stress tolerance in tomato (Xia *et al.*, 2014, Zhou *et al.*, 2014*a*, *b*). The data presented here suggest that that CO_2_-induced stomatal movements are also linked to *RBOH1*-dependent H_2_O_2_ generation in tomato (Figs 3 and 5).

The data presented here show that high CO_2_ concentrations not only increase *RBOH1* transcript levels in leaves but also result in higher H_2_O_2_ accumulation in the roots and leaves, with particular effects in the vascular system (Figs 1 and 3; Supplementary Figs S2, S3, and 5). These high CO_2_–mediated responses were significantly lower in the pTRV-*RBOH1* plants compared with the pTRV controls. The observations presented here show that *RBOH1* is important in the regulation of CO_2_-induced stomatal movements. Moreover, the high CO_2_-dependent alleviation of Na^+^ accumulation and salt-dependent growth inhibition were compromised in the pTRV-*RBOH1* plants grown under high salt. These findings agree with previous results showing that the loss of *AtRBOHF* function in *Arabidopsis* enhanced salt sensitivity (Jiang *et al.*, 2012). Taken together, these results provide evidence in support of the conclusion that growth under high CO_2_ enhances salt stress tolerance by increasing H_2_O_2_-dependent stomatal closure.

Earlier studies have suggested that salt-induced H_2_O_2_ accumulation in the vasculature is also involved in the regulation Na^+^ transport to the shoot (Jiang *et al.*, 2012, 2013). The data presented here show that high atmospheric CO_2_ levels not only increased the H_2_O_2_ in the vascular system but they also exacerbated salt-induced vascular H_2_O_2_ accumulation (Fig. 3; Supplementary Fig. S2B). Moreover, loss of *RBOH1* function in the pTRV-*RBOH1* plants led to lower H_2_O_2_ accumulation in the cells of the vascular tissues under both ambient CO_2_ and high CO_2_ conditions (Fig. 3). The pTRV-*RBOH1* plants exhibited higher Na^+^ accumulation in the vascular cells and in the xylem sap and they showed lower high CO_2_-induced decreases in Na^+^ accumulation in the vascular cells and in the xylem sap (Fig. 7). These results suggest that *RBOH1*-dependent H_2_O_2_ production is critical for the regulation of Na^+^ delivery to the leaves under high CO_2_. Although stomatal conductance values and transpiration rates were substantially decreased after 3 d of salt treatment, the Na^+^ contents of the xylem sap and leaves increased. These findings are consistent with the concept that stomatal movement plays a significant role in CO_2_-induced salt tolerance during the early stages of salt stress, while the rate of Na^+^ delivery via the xylem is more important at the later stages of the high salinity response.

The SOS and NHX families play a major role in maintaining cellular pH values, K^+^ concentrations and Na^+^/K^+^ ratios in order to prevent the perturbations in Na^+^/K^+^ homeostasis caused by high salinity (Zhu *et al.*, 1998; Bassil and Blumwald, 2014; Reguera *et al.*, 2014). Salt-induced increases in the levels of transcripts involved in salt tolerance and in Na^+^ homeostasis were higher in plants grown under high CO_2_ compared with ambient CO_2_ conditions (Fig. 8). Furthermore, the levels of the salt-induced and high CO_2_-induced transcripts were lower in the pTRV-*RBOH1* plants than the pTRV controls, a finding that can be linked to the higher Na^+^:K^+^ ratios in the former than the latter. Taken together, these results suggest that H_2_O_2_ production is involved in salt-induced expression of the *SOS* and *NHX* genes under high CO_2_ conditions. *RBOH*-mediated increases in ROS accumulation are thought to enhance *SOS1* mRNA stability, which contributes to the maintenance of ion homeostasis under salt stress conditions (Chung *et al.*, 2008). In addition to regulating Na^+^ exclusion into the soil, *SOS1* also functions in retrieving Na^+^ from the xylem under high salt stress (Shi *et al.*, 2002). This function may explain the reduced Na^+^ levels observed in the xylem sap of plants grown under high CO_2_. The authors are unaware of any published evidence demonstrating an effect of H_2_O_2_ on NHX expression or activity but it is possible that H_2_O_2_ may regulate NHX expression indirectly via the SOS pathway. It has already been shown that over-expression of *SlSOS2* in transgenic tomato plants confers salt tolerance by up-regulation of NHX genes (Huertas *et al.*, 2012).

The mechanisms by which changes in apoplastic H_2_O_2_ levels regulate the abundance of salt response transcripts are unknown. However, exposure to a range of abiotic stresses, including salt stress, generally induces ABA biosynthesis and accumulation (Tuteja, 2007), which regulates *SOS2* gene expression (Ohta *et al.*, 2003) and activates MAPKs such as MPK6 via RBOH-dependent H_2_O_2_ production (Kovtun *et al.*, 2000; Samuel *et al.*, 2000; Lu *et al.*, 2002; Zhang *et al.*, 2006; Zhang *et al.*, 2007; Xing *et al.*, 2008). The C-terminal region of the *Arabidopsis SOS1* gene is phosphorylated by MPK6 under high NaCl conditions (Yu *et al.*, 2010). It has been previously shown that RBOH1-triggered H_2_O_2_ accumulation activates MPK1/2 in tomato leaves and that silencing of *RBOH1* impairs stress-induced ABA synthesis (Zhou *et al.*, 2014*b*). MAPK3/6 are involved in salinity tolerance in rice (Li *et al.*, 2014). Here it is shown that the levels of *MAPK1/2* and *MAPK3* transcripts, which are homologues of the *Arabidopsis MAPK6* and *MAPK3* genes, respectively, were higher in plants grown under conditions of salinity and high CO_2_ (Fig. 8). These findings suggest that MAPK signalling cascades are involved in the increased expression of SOS genes under these conditions.

High CO_2_-induced ROS accumulation may also increase free cytosolic Ca^2+^ concentrations (Foreman *et al.*, 2003), which may in turn lead to an improved cellular Na^+^/K^+^ balance by enhancing Na^+^ extrusion and maintaining K^+^ influx (Demidchik *et al.*, 2002; Zhu, 2002; Munns and Tester, 2008). These factors may also contribute to the higher tolerance to salt stress that was observed under high CO_2_ conditions in this study. Moreover, an increase in *RBOH*-dependent H_2_O_2_ production has been proposed as a mechanism to increase HKT-mediated Na^+^ unloading from the xylem sap (Jiang *et al.*, 2012). In turn, this process may trigger an antioxidant response to police ROS metabolism and prevent further increases in ROS accumulation, resulting in a further mitigation of the negative impacts of salt stress (Ben *et al.*, 2014).

In summary, the findings reported here demonstrate that high CO_2_ concentrations can counteract the negative impact of salt stress on photosynthesis and biomass production in an apoplastic H_2_O_2_-dependent manner in tomato plants. The regulation of apoplastic H_2_O_2_ levels also makes a major contribution to the regulation of Na^+^ transport from roots to shoots, a process that is influenced by stomatal movement and by Na^+^ delivery from the xylem to leaf cells. Regulated changes in apoplastic H_2_O_2_ levels may therefore play an important role in triggering the mechanisms that underpin salt tolerance and associated detoxification pathways.

## Supplementary data

Supplementary data are available at *JXB* online.

Table S1. Gene-specific primers designed for qRT-PCR.

Fig. S1. Gas exchange in tomato plants in response to salt treatments and elevated CO_2_ levels.

Fig. S2. H_2_O_2_ accumulation in response to salt treatments and elevated CO_2_ levels.

Fig. S3. Quantification of H_2_O_2_ in salt-treated plants under ambient and elevated CO_2_ levels.

Fig. S4. Relative RBOHs transcript abundance and NADPH oxidase activity in the leaves from *RBOH1*-silenced tomato plants.

Fig. S5. Quantification of H_2_O_2_ in salt-treated pTRV control and *RBOH1* silenced (pTRV-*RBOH1*) tomato plants under ambient and elevated CO_2_ level.

Supplementary Data
