# Supplementary material for: High atmospheric carbon dioxide-dependent alleviation of salt stress is linked to RESPIRATORY BURST OXIDASE 1 (RBOH1)-dependent H2O2 production in tomato (Solanum lycopersicum)
Source: J Exp Bot. 2015 Sep 28;66(22):7391–404. doi: 10.1093/jxb/erv435 (PMC4765801; doi:10.1093/jxb/erv435)
Supplement: Supplementary Data [file supp_66_22_7391__index.html]

High atmospheric carbon dioxide-dependent alleviation of salt stress is linked to RESPIRATORY BURST OXIDASE 1 (RBOH1)-dependent H2O2 production in tomato (Solanum lycopersicum) — High atmospheric carbon dioxide-dependent alleviation of salt stress is linked to RESPIRATORY BURST OXIDASE 1 (RBOH1)-dependent H2O2 production in tomato (Solanum lycopersicum) — Supplementary Data 

# High atmospheric carbon dioxide-dependent alleviation of salt stress is linked to RESPIRATORY BURST OXIDASE 1 (*RBOH1*)-dependent H2O2 production in tomato (*Solanum lycopersicum*)

## Supplementary Data

Data files

- Supplementary Data - Supplementary Data
