# Supplementary material for: High atmospheric carbon dioxide-dependent alleviation of salt stress is linked to RESPIRATORY BURST OXIDASE 1 (RBOH1)-dependent H2O2 production in tomato (Solanum lycopersicum)
Source: J Exp Bot. 2015 Sep 28;66(22):7391–404. doi: 10.1093/jxb/erv435 (PMC4765801; doi:10.1093/jxb/erv435)
Supplement: Supplementary Data [file supp_erv435_jexbot153015_file001.pdf]

High atmospheric carbon dioxide-dependent alleviation of salt stress is linked to RBOH1-dependent H_2O_2 production in tomato (*Solanum lycopersicum*).

Changyu Yi, Kaiqian Yao, Shuyu Cai, Huizi Li, Jie Zhou, Xiaojian Xia, Kai Shi, Jingquan Yu, Christine Helen Foyer & Yanhong Zhou

SUPPLEMENTARY DATA

Table S1. Gene-specific primers designed for qRT-PCR.

Gene	Accession numbers	Forward primer	Reverse primer
Actin2	Solyc03g078400	TGGTCGGAATGGGACAGAAG	CTCAGTCAGGAGAACAGGGT
RBOH1	Solyc08g081690	TCCAGCACAAGATTACCG	CCTCCATTGCGACGAT
SOS1	Solyc01g005020	TCGAGTGATGATTCTGGTGG	GAGCCTTTCCACACTGTGAT
SOS2	Solyc12g009570	TCTATCCGCTTTGTTTG	ATTGACCAGCCCTATTT
SOS3	Solyc03g083320	CCACCCAAATGCACCAGTAG	CAGCGCCAAAACCATCTCTT
NHX1	Solyc06g008820	GTGGTGTTTGGGCTGAT	TTGCTCGTTAGTGAGAAGTG
NHX2	Solyc04g056600	CCTTTGAGGGGAACAATGG	CATCTTCATCTTCGTCTCC
NHX3	Solyc01g067710	CTCAAGAGTCACCACCAAGCA	CCAACCAAAACAAGACCCAACA
MAPK1	Solyc12g019460	TGCACCTCCGGTCAACAA	GGCAGTGCTCCTCAGATAAA
MAPK2	Solyc08g014420	AGGGTTTACTATTTACGG	TGGAGGCTTATACTTCG
MAPK3	Solyc06g005170	CTAAATTTCTATCAATAATGGTTGATGC	GCGGAGGAATCACATCTCTT
WFI1	Solyc03g117980	ATACATCTTCGTCTACCT	ACAATAGTTTCTTCGTG
SlRBOHA	Solyc01g099620	GAGAGTAGGATTCAGCGGT	GCCTCTTTTCGAGCTTGCT
SlRBOHC	Solyc05g025680	AGAAACGTCGGAGCATG	AGAAACGTCGGAGCATG
SlRBOHD	Solyc06g068680	TACACCACCAAATCTAACG	TGCCCAGTGCTTCAATC
SlRBOHE	Solyc06g075570	GGGTCCTAACATTGTGGTTGT	AGAGTCTCTCCTGCACCTTTA
SlRBOHF	Solyc07g042460	GCAGCTCAAAACGATCC	CATTGCTCCTCCGACAT
SlRBOHH	Solyc11g072800	CATGTGTGACAAGAATGGTGATG	TGCTGCATGTTTCTTGAAGTTT

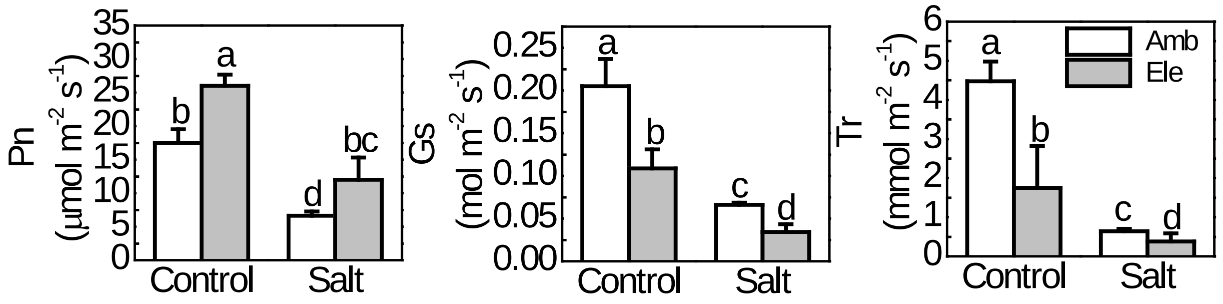

Figure S1. Gas exchange in tomato plants in response to salt treatments and elevated CO_2 levels. The data were measured 3 d after salt treatment. The data values are the means ± SD of four replicates. Means denoted by the same letter did not differ significantly according to Tukey's test ($P < 0.05$).

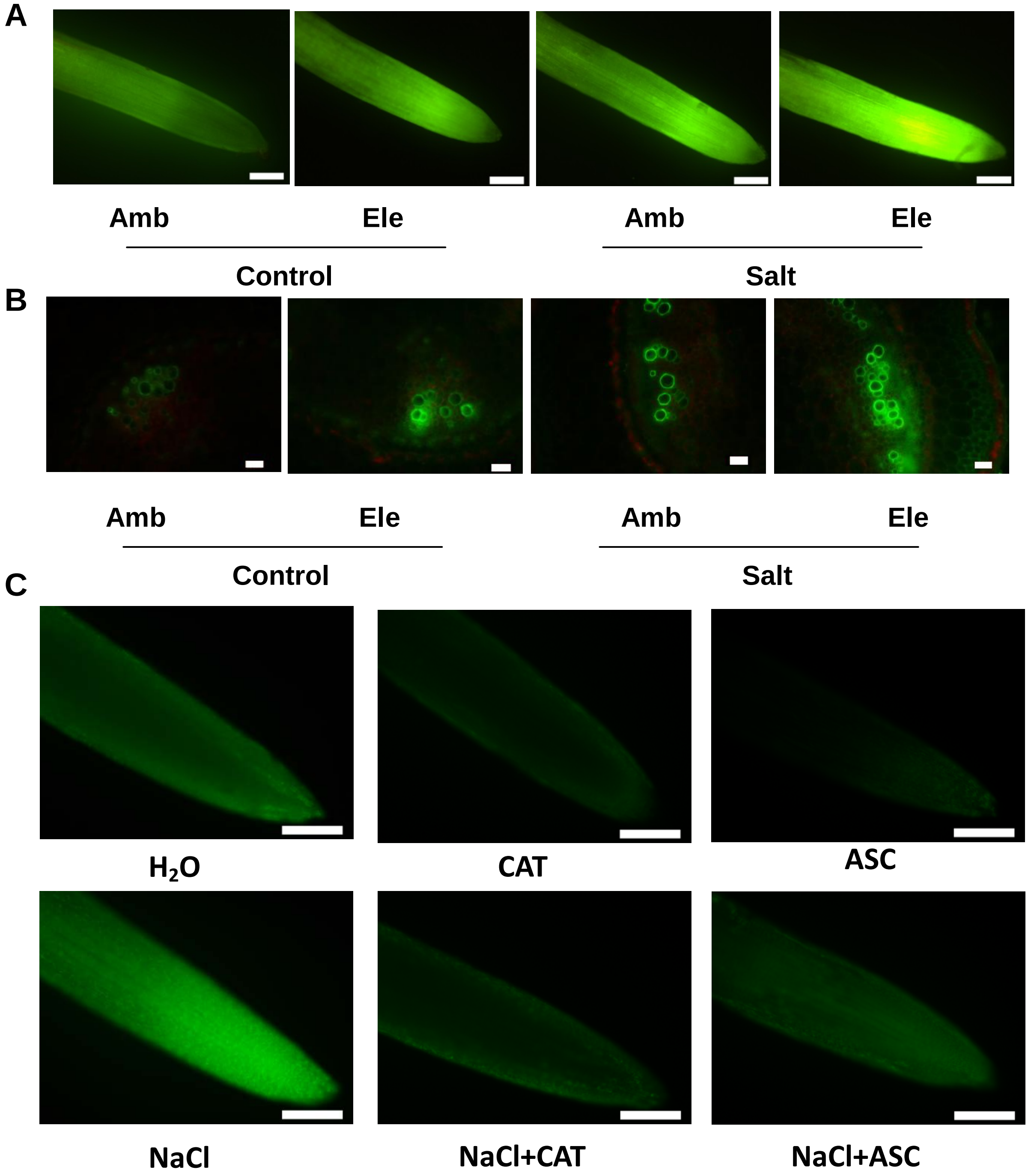

Figure S2. H_2O_2 accumulation in response to salt treatments and elevated CO_2 levels. (A) H_2O_2 accumulation in roots detected by 2,7-dichlorofluorescein diacetate (H_2DCF-DA). (B) H_2O_2 accumulation in petiole. Samples were imaged on day 3 after salt treatment. (C) H_2O_2 accumulation in roots with or without CAT and ASC. The root segments were incubated with 1 mM ascorbate (ASC) or 100 U ml^{-1} catalase (H_2O_2 scavenger) for 30 min before exposure to H_2DCF-DA.

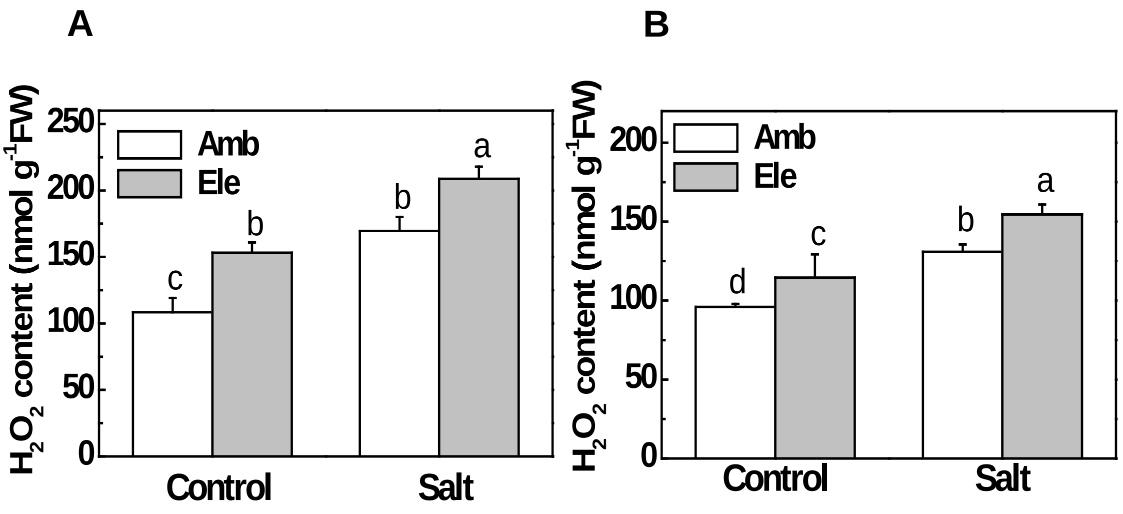

Figure S3. Quantification of H_2O_2 in salt treated plants grown under elevated (760 μmol mol^{-1}) or ambient CO_2 (380 μmol mol^{-1}) conditions. (A) Leaves. (B) Roots. Plant samples were taken at 7 d after exposure to salt. The data values are the means ± SD of four replicates. Means denoted by the same letter did not differ significantly according to Tukey's test ($P < 0.05$).

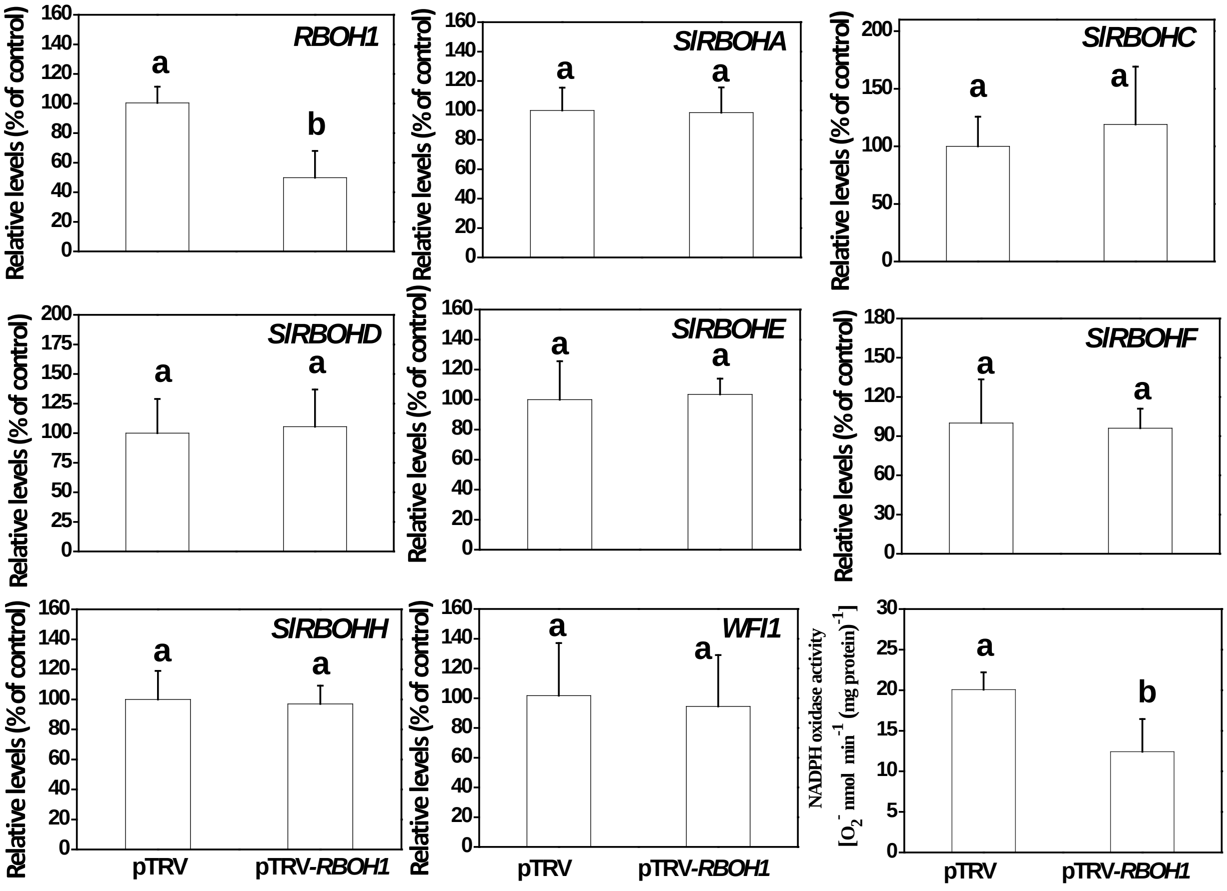

Figure S4. Relative *RBOHs* transcript abundance and NADPH oxidase activity in the leaves from *RBOH1* silenced tomato plants. The 5th leaf at 8-leaf stage was used. Data values are the means ± SD of eight replicates. The levels are presented as percentages of the mean levels in control pTRV plants, which were set to 100%.

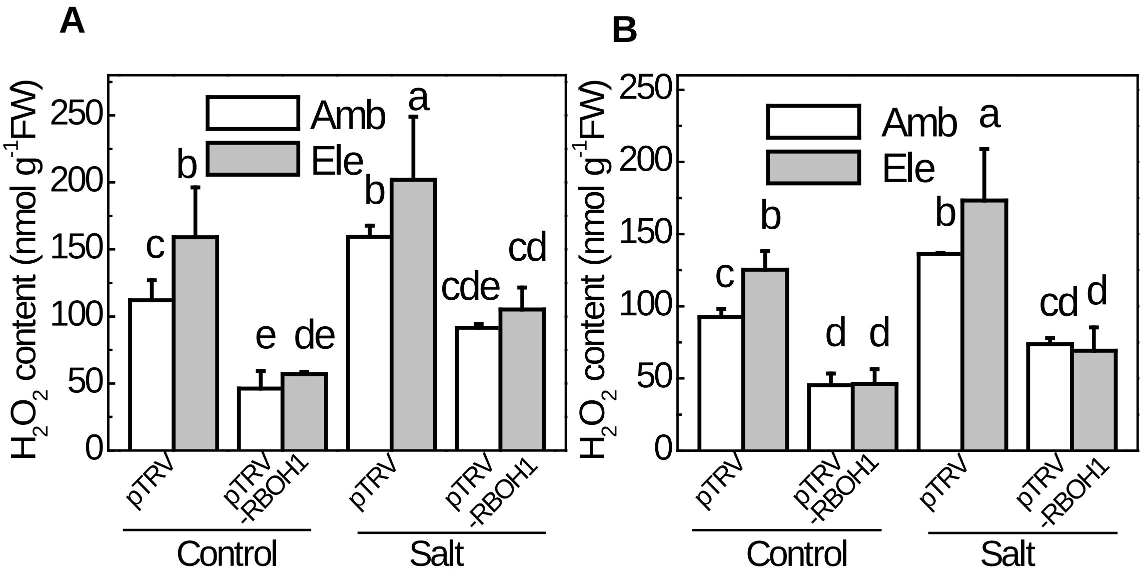

Figure S5. Quantification of H_2O_2 in salt treated pTRV empty vector control and *RBOH1* silenced tomato plants grown under elevated (760 μmol mol^{-1}) or ambient CO_2 (380 μmol mol^{-1}) conditions. (A) Leaves. (B) Roots. Plant samples were taken at 7 d after exposure to salt. The data values are the means ± SD of four replicates. Means denoted by the same letter did not differ significantly according to Tukey's test ($P < 0.05$).
